# Composites Based on PLA/PHBV Blends with Nanocrystalline Cellulose NCC: Mechanical and Thermal Investigation

**DOI:** 10.3390/ma17246036

**Published:** 2024-12-10

**Authors:** Patrycja Bazan, Arif Rochman, Krzysztof Mroczka, Kamil Badura, Mykola Melnychuk, Przemysław Nosal, Aleksandra Węglowska

**Affiliations:** 1 Faculty of Materials Engineering and Physics, Cracow University of Technology, 31-155 Krakow, Poland; krzysztof.mroczka@pk.edu.pl; 2 Department of Industrial and Manufacturing Engineering, University of Malta, 2080 Msida, Malta; arif.rochman@um.edu.mt; 3 CUT Doctoral School, Faculty of Materials Engineering and Physics, Cracow University of Technology, 31-155 Krakow, Poland; kb@shmsystem.pl; 4 Department of Materials Science, Lutsk National Technical University, Lvivska 75, 43018 Lutsk, Ukraine; m.melnychuk@lntu.edu.ua; 5 Department of Machine Design and Maintenance, AGH University of Krakow, 30-059 Krakow, Poland; pnosal@agh.edu.pl; 6 Łukasiewicz—Upper Silesian Institute of Technology, The Welding Centre, Bł. Czesława, 44-100 Gliwice, Poland; aleksandra.weglowska@git.lukasiewicz.gov.pl

**Keywords:** biocomposites, PLA/PHBV blends, nanocrystalline cellulose, accelerated aging, mechanical properties, thermal properties

## Abstract

This study investigates the physical and mechanical properties of biodegradable composites based on PLA/PHBV blends modified with different content of nanocrystalline cellulose (NCC) of 5, 10, and 15 wt.%. Density measurements reveal that the density of the composite increases with increasing NCC content. Water absorption tests demonstrate a gradual increase in the composite water content with increasing incubation time, reaching stabilization after approximately 30 days. Mechanical testing was also carried out on both on conditioned samples after the process of hydrolytic degradation and accelerated thermal aging. The conditioned composites show an increase in the stiffness of the materials with increasing content of nanocrystalline cellulose. The ability to deform and the ability to absorb energy when the sample is dynamically loaded decrease. The repeated strength tests, after the process of incubation of samples in water and after the process of accelerated thermal aging, show the degradation of composite materials; however, it is noticed that the introduction of cellulose addition reduces the impact of the applied artificial environment in aging tests. The findings of this study indicate promising applications for these types of materials, characterized by high strength and biodegradability under appropriate conditions. Household items such as various containers or reusable packaging represent potential applications of these composites.

## 1. Introduction

Plastics are pervasive due to their affordability, durability, and customizable textures and colors, meeting both functional and aesthetic needs. However, their widespread use has led to environmental problems from non-recyclable waste accumulation and slow decomposition. Petroleum-based materials also cause environmental harm throughout their life cycle. Consequently, research is increasingly focused on developing renewable and natural source materials with comparable properties that degrade harmlessly and quickly post-use, aiming to reduce pollution and replace non-renewable materials [1,2,3]. 

Polymer blends are materials created by mixing different polymers to enhance properties or replicate existing materials at reduced costs and higher profitability [4,5]. Blends are typically made through mechanical mixing or synthesis in the same medium. Creating a blend necessitates choosing ingredients with an appropriate miscibility. When the miscibility is insufficient, an additive like a compatibilizer can aid mixing. Blends of various polymers have diverse applications due to their distinct properties, such as substance carriers, electrically conductive materials, or various membranes [6,7].

PLA/PHBV blends have been explored as fiber-reinforcement materials in textiles and as finished products or composite matrices. Pure PLA fibers have limitations like poor heat resistance and short shelf life, causing hardening and brittleness with temperature and storage. Adding PHBV to PLA improves flexibility and reduces shrinkage, making the PLA/PHBV blend a promising alternative for textile applications [8,9]. The PLA/PHBV blend is also used in food packaging. Hernández-García examined how phenolic acids affect the film properties of the blend, finding that protocatechuic acid raised PLA’s glass transition temperature and caused an undercooling effect in PHBV. Additionally, the acid improved stiffness and barrier properties against oxygen and water vapor and phenolic acids demonstrated antibacterial activity against certain bacterial strains [10,11]. 

Recent studies emphasize biodegradable matrices in polymer composites to address environmental issues and the need for sustainable materials. Research on cellulose fiber reinforcement in PBAT and PLA/PBAT blend matrices evaluated their strength and water resistance. Findings indicated uniform water absorption for all configurations. Unaltered PBAT and PLA/PBAT blends showed decreased strength and elongation at break, yet their elastic modules were enhanced. Surface modification techniques were identified as promising for improving material properties [12].

Studies have documented polylactide (PLA) composites for their biodegradability, often combined with natural fibers for environmental benefits. Tests used PLA as the matrix, linen as reinforcement, and triacetin as a plasticizer, produced via pressure molding with a twin-screw mixer, containing 30% and 40 wt.% fiber. The results were compared to polypropylene (PP) composites reinforced with flax fibers. PLA composites showed a significant improvement over PP composites used in the automotive industry. A fiber addition notably enhanced PLA’s stiffness, though natural fiber adhesion to the polymer matrix was problematic. The plasticizer was ineffective and even detrimental [13].

Oil palm cellulose fibers were incorporated into a PLA matrix to create a biocomposite with improved thermal properties. Higher cellulose fiber content increased the Young’s modulus but reduced tensile strength and elongation at break, likely due to the poor fiber dispersion that was observed microscopically. Optimizing fiber dispersion and adhesion is anticipated to improve mechanical strength [14]. 

PHBV, like PLA, has not only biodegradability but also biocompatibility. It is a polyhydroxyalkanoate polymer, which is a non-toxic, biocompatible plastic that is produced naturally by bacteria, making it an excellent alternative to many non-biodegradable synthetic polymers [15,16,17]. A composite material was created by integrating coconut fibers with PHBV. To enhance fiber–matrix adhesion, the fibers were surface-treated. Conventional and micro-injection molding techniques were employed. The results indicated that the strength of both conventional and micro-samples improved with the addition of treated and untreated fibers. Sialane-treated composites exhibited the highest strength and elongation at break. Furthermore, incorporating fibers into PHBV increased the composite’s crystallinity, irrespective of fiber modification [18]. 

Nanocrystalline cellulose (NCC) is gaining prominence in scientific research, particularly due to its capacity to enhance the mechanical properties of biodegradable polymer matrices. The incorporation of NCC into such materials can substantially increase the tensile strength and elastic modulus, as evidenced by studies on polymers such as polylactic acid (PLA) and poly(butylene succinate) (PBS) [19,20]. Specifically, the addition of NCC to PBS results in a significant increase in both strength and elongation at break, attributed to the uniform dispersion of nanofillers and the strong adhesion between NCC and the polymer matrix [20]. The mechanical reinforcement provided by NCC is also associated with their high aspect ratio and crystallinity, which facilitates efficient load transfer in the polymer matrix [21,22].

In addition to enhancing mechanical properties, NCCs also contribute to increasing the thermal stability of biodegradable polymers. They function as nucleating agents that promote crystallization in the polymer matrix, resulting in higher thermal stability [23,24]. For instance, in poly(hexamethylenesuccinate) composites, the presence of NCCs not only improves mechanical properties but also increases the crystallization rate, which translates into enhanced thermal properties [24]. This characteristic is particularly advantageous in applications requiring high thermal stability.

Incorporating NCCs into biodegradable polymers significantly impacts biodegradation. Research shows that NCCs enhance water absorption, facilitating the hydrolytic degradation of the polymer matrix and promoting polymer chain disintegration. For example, in PLA composites, NCCs improve water penetration, accelerating polymer hydrolysis [25]. However, it is important to note that excessive NCC content can potentially reduce the biodegradability of the material due to the formation of strong hydrogen bonds between NCCs and the polymer, which may impede the access of microorganisms to the matrix [26].

Surface modification of NCCs, such as grafting with hydrophobic polymers like poly(lactic acid), enhances their dispersion in non-polar matrices, improving composite properties [27]. This study examined incorporating nanocellulose into PHBV to assess filler influence on material properties. Cellulose nanocrystals (NCCs) and cellulose nanofibrils (CNFs) were used, both enhancing thermal stability, with CNFs showing significant improvement. Both cellulose types increased strength compared to pure PHBV, with the optimal configuration being an NCC composite containing 1 wt.% fiber [27]. 

Polymer blends are increasingly used as composite matrices, allowing for the customization of composite properties by adjusting the reinforcement and polymer matrix types. A notable example is the PLA/PHBV blend with carbon nanotubes (CNTs) for electrical and electromagnetic applications. This composite includes three components: PLA as an inexpensive base material, PHBV for enhancing mechanical properties, and CNTs for imparting electrical and electromagnetic properties. Unlike natural fibers, CNTs adhere well to the matrix and distribute uniformly. Samples with 0.5 wt.% and 1 wt.% CNT content showed promising results, with the higher CNT content material demonstrating significant damping of electromagnetic vibrations, suggesting diverse potential applications [28]. Polymer blends with a 30:70 ratio of PLA to PHBV and nanocrystalline cellulose (NCC) from palm waste showed improved properties with 0.25 wt.% NCC compared to the pure blend. However, excessive cellulose content negatively affected blend performance by restricting polymer chain mobility. The elastic modulus decreased at 0.5 wt.% NCC, and flexural strength reduced at 1 wt.% NCC. These results emphasize the need for an optimal blend and NCC proportions for effective packaging applications [29].

This study evaluates the feasibility of manufacturing and using biodegradable composites of polylactic acid (PLA) and poly(3-hydroxybutyrate-co-3-hydroxyvalerate) (PHBV) modified with nanocrystalline cellulose (NCC) particles for injection-molded components. It is hypothesized that incorporating NCC particles, due to their crystal structure and size, will enhance the mechanical properties, durability, and stability of the composites. The size variations in cellulose fibers, dependent on their form and origin, result in distinct properties allowing diverse applications. The production and use of cellulose are expected to increase in various industries, such as aviation, automotive, construction, and filter production [30]. Recently, there has been a focus on natural fibers because of their abundance, renewable nature, and diverse forms. Natural fibers are eco-friendly, derived from sustainable resources, decomposable by the environment, and minimally impact the surroundings during storage or recycling at the end of their life cycle.

## 2. Materials and Methods

### 2.1. Materials

The matrix material selected was a PLA/PHBV polyester blend containing 90% PLA and 10% PHBV, ColorFabb (Belfeld, The Netherlands). Nanocrystalline cellulose (NCC) by Nanografi Nano Technology (Jena, Germany) was used as the reinforcement material with particle diameter and length of 10–20 nm and 300–900 nm, respectively. Polymer composites containing 5, 10, and 15 wt.% of nanocrystalline cellulose were produced. The designation and description of the manufactured materials are shown in Table 1. The tensile testing specimens were prepared based on the European standards EN ISO 3167 in variant A [30], whereas PN EN ISO 14125 [31] standard was used to produce the specimens for three-point bending tests. The PLA/PHBV and its composites were made by injection molding (Krauss Maffei KM 40-125 C1, KraussMaffei Group GmbH, Monachium, Germany), with parameters listed in Table 2. 

### 2.2. Methods of Testing

The main desirable composite properties are not only their low density and required strength but also their change in properties due to the external environment. The density of the composites was determined by the hydrostatic method using a RADWAG WAS 22W laboratory scale (Radom, Poland). 

Water absorption was conducted by the gravimetric method following the PN-EN ISO 62:2008 standard [32]. The weight was measured using an electronic Ohaus Adventurer laboratory balance (Parsippany, NJ, USA). After weight measurements, the water absorption N_w_ was calculated from Formula (1):

(1) $$N_{w}=\frac{m_{2}-m_{1}}{m_{1}}\cdot 100\%$$

where

N_w_—the weight water absorption, %;
m_1_—mass of the sample before being placed in the solution, g;
m_2_—weight of the sample after being removed from the solution and dried with paper towels, g.

Hydrostatic degradation tests were also carried out. Samples were immersed in distilled water at 21 °C. The trial was conducted over four weeks for both water absorption and hydrothermal degradation. After water absorption and hydrothermal degradation, mechanical tests were performed. Basic strength tests are critical because they indicate the direction for further research. Both static tension and static bending tests were performed. Impact testing was conducted on unnotched specimens using the Charpy method using a Zwick/Roell HIT5.5P hammer (Ulm, Germany) according to PN-EN ISO 179-1:2010 [33]. The static tensile test (PN-EN ISO 527-1:2010) [34] and the static bending test (PN-EN ISO 178:2011) [35] were performed on a Shimadzu AGS-X 10 kN test machine (Kyoto, Japan. load capacity: 1 N to 10 kN; test speed: 0.001 to 1000 mm/min; test force accuracy: within ±0.5% of display test force (for 1/1 to 1/500 of load cell capacity)) with a test speed of 10 mm/min. Strength tests were carried out on at least 5 samples. All strength tests were repeated after the hydrolytic degradation process and after the accelerated thermal aging process. An aging chamber was used for accelerated thermal aging, simulating damage from sunlight and rain that could occur in atmospheric conditions over several months or years. Samples are subjected to alternating UV radiation and moisture cycles under elevated temperature conditions to perform this test. Types of damage may include changes in sample surface color, cracks, brittleness, loss of strength, and oxidation. Aging tests were carried out following the cycle presented in Table 3. This cycle was repeated over 1000 h.

The first mechanical hysteresis loops were determined to visualize the viscoelastic nature of the produced composites. The test was carried out in two different approaches. The first approach included an applied load of up to 60% of the maximum force needed to break the sample determined during the tensile test. This approach aimed at determining the displacement that occurs during cyclic operation. In the second approach, a force that is assumed to cause a displacement value of 1 mm was applied. This approach aimed to register changes in the required force to perform the displacements. The tests were carried out on a Shimadzu AGS-X 10 kN testing machine (Kyoto, Japan) with software that allows energy dispersion analysis (Autograph Trapezium X v. C224-E048). The loading and unloading speed was 100 mm/min. 

Fatigue tests were carried out using a standard electrohydraulic servo fatigue testing machine Shimadzu EHF-E Series (Kyoto, Japan). The research was conducted based on the assumptions of the Lehr method. This method was initially used for fatigue testing of metals as an alternative to the long-term Wohler method [36]. The Lehr method uses the observation that a sudden increase in the dissipation energy, strain, and temperature of the tested samples accompanies fatigue failure. The essence of the method is to determine these parameters in an accelerated fatigue test under cyclic loading conditions with gradually increasing amplitude and present them as a function of the maximum stress. This method was helpful in determining fatigue strength as a comparative parameter that describes the fatigue properties of polymer composites with a thermoplastic matrix [37].

To fully understand the effect of the NCC contents on the thermal behavior of the composites, a differential scanning calorimeter (DSC) DSC3+ and a dynamic mechanical analyzer (DMA) DMA 1 (both Mettler Toledo, Greifensee, Switzerland) were used. The DSC sample weight was determined using a MYA 11.4Y Plus microbalance (RADWAG, Radom, Poland). All DSC samples were then subjected to heating from 20 °C to 200 °C, then cooling from 200 °C to 20 °C, and finally heating again from 20 °C to 200 °C, all of which were carried out with a heating and cooling rate of 2 K/min. The DSC analysis was conducted under a nitrogen atmosphere with a flow rate of 60 mL/min. Apart from determination of phase transition temperatures such as glass transition and melting temperature, crystallinity degree (X_c_) was calculated using Equation (2), where ΔH_m_ is the melting enthalpy, ΔH_cc_ is the cold crystallization enthalpy, and ΔH_m_,100% is the theoretical melting enthalpy of PLA with a value of 93 J/g [38].

(2) $$X_{c}=\frac{{∆H}_{m}-{∆H}_{cc}}{{∆H}_{m,100\%}}$$

Regarding the DMA, all measurements were carried out in tension mode between 20 °C and 80 °C, with a displacement of 10 µm and a frequency of 1 Hz. 

Microscopic observations were performed using a JEOL JSN5510LV scanning electron microscope (JEOL Ltd., Tokyo, Japan). Before testing, the sample surface was covered with a conductive gold layer on a JOEL JEE-4X vacuum evaporator (JEOL Ltd., Tokyo, Japan).

## 3. Results and Discussion

### 3.1. Thermal Analysis

**Figure 1:**
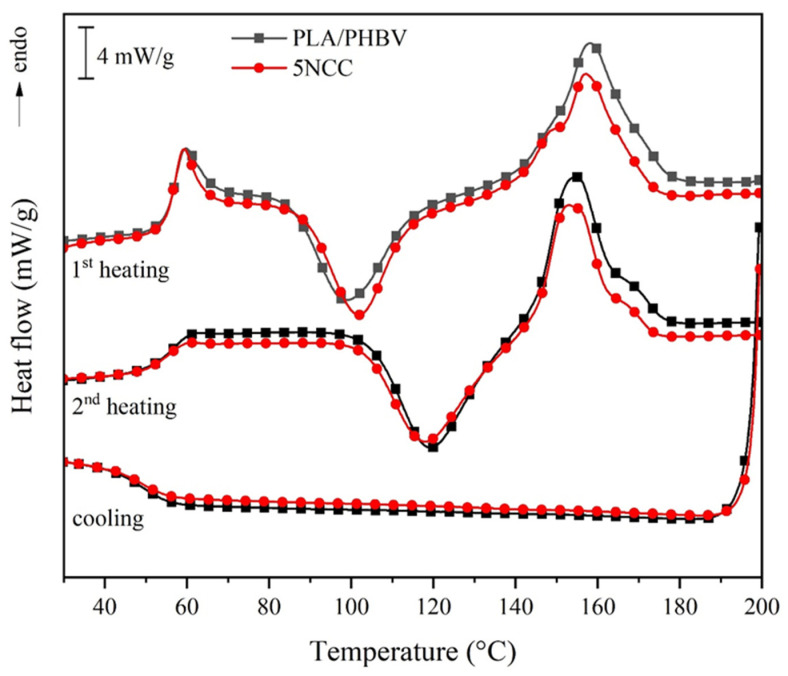
DSC thermograms of PLA/PHBV and 5NCC specimen.

Figure 1 shows the DSC results of the specimens molded from the pure PLA/PHBV and the PLA/PHBV/NCC composite with a 5 wt.% NCC content. The latter serves as a representative sample due to the similarity in DSC curve progression among the PLA/PHBV/NCC composites. The first heating curve reveals the specimens’ thermal history resulting from the molding process. The exothermic peak between 85 °C and 120 °C indicates post-crystallization or cold crystallization. The bigger the area of the post-crystallization peak, the fewer crystallites already formed during the injection molding process. The endothermic peak between around 140 °C and 180 °C is associated with the melting process of all crystallites formed during both injection molding and DSC cold crystallization. 

Since the thermal history of the sample has been removed in the first heating cycle, the cooling cycle shows solely the crystallization behavior of the material. However, it is worth noting that the DSC cooling rate of 2 K/min is significantly different than the one in the injection molding process. The cooling curves of both samples did not show any exothermic peak, i.e., no crystallization occurs during the transition from a melted to a solid state. The crystallites were then fully formed during the cold crystallization process in the second heating cycle. In all DSC curves, the glass transition temperature is indicated by this step, in this case at around 55 °C, due to the change in the sample’s heat capacity. Comparable results have been observed in other studies. The thermal characteristics of PLA and PHBV composites, particularly those incorporating nanocrystalline cellulose (NCC), were examined utilizing differential scanning calorimetry (DSC). Analyses reveal that an exothermic peak within the range of 85–120 °C (on the initial heating curve) indicates post-crystallization or cold crystallization, wherein a greater peak area signifies a lower degree of crystallization during formation [39,40]. The endothermic peak observed in the temperature range of 140–180 °C corresponds to the melting of crystallites. The absence of an exothermic peak on the cooling curve (2 K/min) indicates that crystallization predominantly occurs during cold crystallization in the subsequent heating cycle [41,42]. The glass transition temperature (T_g_) is approximately 55 °C, which indicates the thermal stability of the composites [43]. NCC enhances nucleation activity, facilitates crystallization, and improves thermal stability through interactions with the polymer matrix [44,45]. Furthermore, NCC augments the mechanical properties of composites by increasing stiffness and enhancing interfacial bonding [40,46]. 

**Figure 2:**
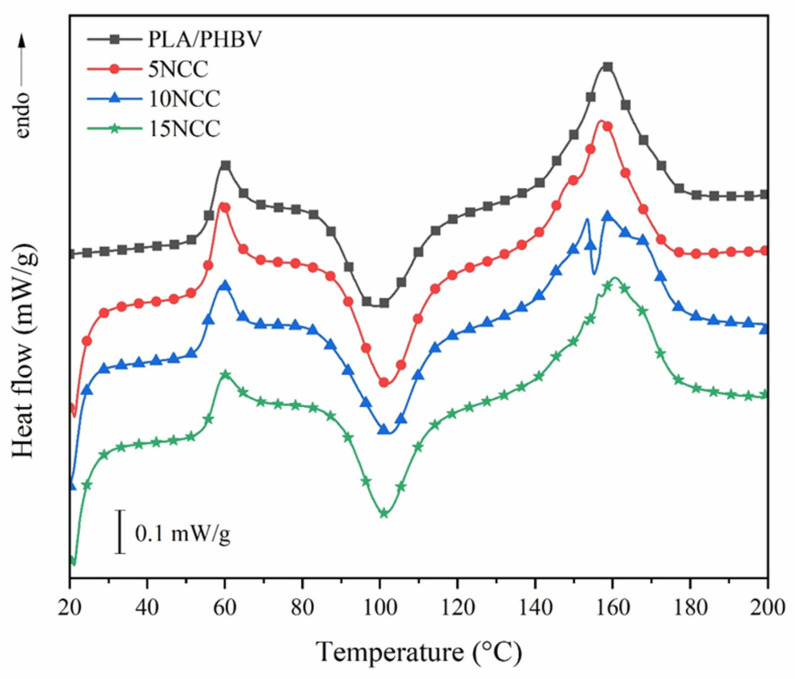
First heating curve of the PLA/PHBV and PLA/PHBV/NCC molded.

The DSC results, as shown in Figure 2 and Table 4, revealed that there is no significant trend and difference in terms of the glass transition temperature (T_g_), melting temperature (T_m_), and crystallization degree when NCC is added to the PLA/PHBH blend up to 15 wt.%. The 15NCC samples show the highest degree of crystallization of around 11%. However, their standard deviation value is also relatively higher than the one of the samples with a lower NCC content. Thus, all values could be considered as not significantly different. However, it could also be observed that the addition of NCC causes the formation of an additional melting peak at around 150 °C. This indicates that another crystallite domain with a lower melting temperature is formed. This additional peak becomes slightly bigger in size with a higher NCC content.

As described in the method, the crystallinity degree (X_c_) is calculated by dividing the difference between the melting enthalpy ΔH_m_ (the endothermic peak at around 159 °C) and the cold crystallization enthalpy ΔH_cc_ (the exothermic peak at around 100 °C) by the theoretical melting enthalpy of PLA (ΔH_m_,100%), with a value of 93 J/g. Therefore, those injection-molded materials can be considered as predominantly amorphous materials as their crystallization degree is relatively low. However, if, e.g., through the annealing process, the cold or post-crystallization could completely occur, a crystallization degree of around 34% could be obtained (ΔH_m_ directly divided by ΔH_m_,100%). 

**Figure 3:**
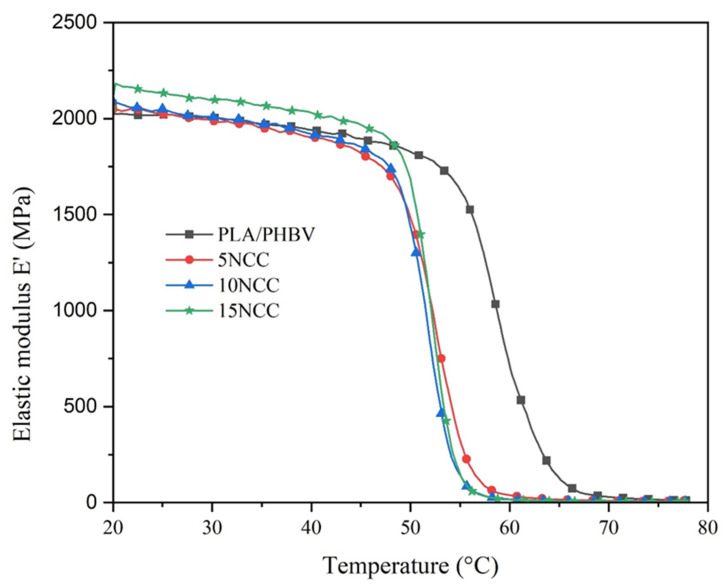
DMA thermograms of the PLA/PHBV and PLA/PHBV/NCC molded specimens.

From the DMA results shown in Figure 3 and Table 5, it can be observed that, in contrast to the DSC results, there is a significant change in the glass transition temperature with the addition of NCC to PLA/PHBV. The discrepancy between DMA and DSC measurements of the glass transition temperature (T_g_) arises from their different testing method. While DSC measures heat flow and detects the glass transition temperature by the change in heat capacity, DMA applies mechanical stress and measures changes in mechanical properties and thus detects the glass transition temperature through alterations in the modulus. In contrast to DSC, DMA is more sensitive to molecular motion so that it can detect changes in glass transition temperatures due to the NCC addition, as shown in these results.

Regardless of the content, the NCC addition reduces the glass transition temperature of pure PLA/PHBV from around 60 °C to around 53 °C. This glass transition temperature reduction is related to the increased mobility of the macromolecules. Since the DSC results revealed that the crystallinity degree of all specimens is relatively low and did not significantly change with the NCC addition, it can be assumed that the increased mobility of the macromolecules occurred mainly in the amorphous domains. In the amorphous domains of the PLA/PHBV/NCC specimens, the NCC particles could act as plasticizers that disrupt the intermolecular forces between the macromolecules, which increases their mobility. This increased mobility lowers the temperature at which the polymer transitions from a glassy to a rubbery state. However, the addition of NCC up to 15 wt.% did not yet significantly change the molecules’ mobility.

Other studies also demonstrated variations in results. This fundamental difference in measurement sensitivity leads to varying Tg values, particularly when additives such as nanocrystalline cellulose (NCC) are incorporated into polylactic acid (PLA) and poly(3-hydroxybutyrate-co-3-hydroxyvalerate) (PHBV) blends. Specifically, the incorporation of NCC has been observed to decrease the Tg of pure PLA/PHBV from approximately 60 °C to approximately 52 °C, which corresponds to increased molecular mobility within the amorphous regions of the polymer matrix. This phenomenon suggests that NCC functions as a plasticizer, effectively reducing intermolecular forces and facilitating greater macromolecular movement [44,47]. Research indicates that the incorporation of NCC not only affects the Tg but also influences the overall thermal and mechanical properties of the PLA/PHBV composites. Specifically, the presence of NCC can enhance the degree of crystallinity in PLA, which is frequently associated with improved mechanical performance due to the nucleating effect of NCC [44,48]. Nevertheless, it is noteworthy that the impact of NCC on mobility becomes limited at concentrations exceeding 15 wt.%, indicating a threshold beyond which additional NCC does not significantly alter the material properties [49,50]. This observation aligns with findings that demonstrate a complex interplay between the components in PLA/PHBV blends, wherein the addition of PHBV can result in a decrease in T_g_, thus indicating a reduction in the rigidity of the polymer matrix [45,51]. Moreover, the mechanical properties of these biocomposites are further influenced by the compatibility and dispersion of NCC within the PLA/PHBV matrix. Studies have shown that improved dispersion of NCC can lead to enhanced mechanical properties, while poor compatibility may result in reduced performance [52]. Moreover, the mechanical properties of these biocomposites are substantially influenced by the compatibility and dispersion of NCC within the PLA/PHBV matrix. Empirical studies have demonstrated that enhanced dispersion of NCC can result in improved mechanical properties, whereas poor compatibility may lead to diminished performance [53]. Consequently, the combined effects of NCC functioning as a plasticizer and nucleating agent, in conjunction with the inherent properties of PLA and PHBV, contribute to the observed variations in Tg and mechanical behavior within these biocomposite systems.

These results also mean that products made from these PLA/PHBV/NCC composites have a lower thermal stability compared to the ones made from the PLA/PHBV blend. At room temperature (20 °C), the elastic modulus of all specimens increases slightly with the increasing NCC content. Up to around its glass transition temperature, the elastic modulus of 15NCC composite always has the highest stiffness. Due to the lower glass transition temperature of the PLA/PHBV/NCC composites, their stiffness starts to drop significantly at around 50 °C, while for the pure PLA/PHBV, it occurs at around 55 °C.

### 3.2. Physical and Mechanical Examination

**Figure 4:**
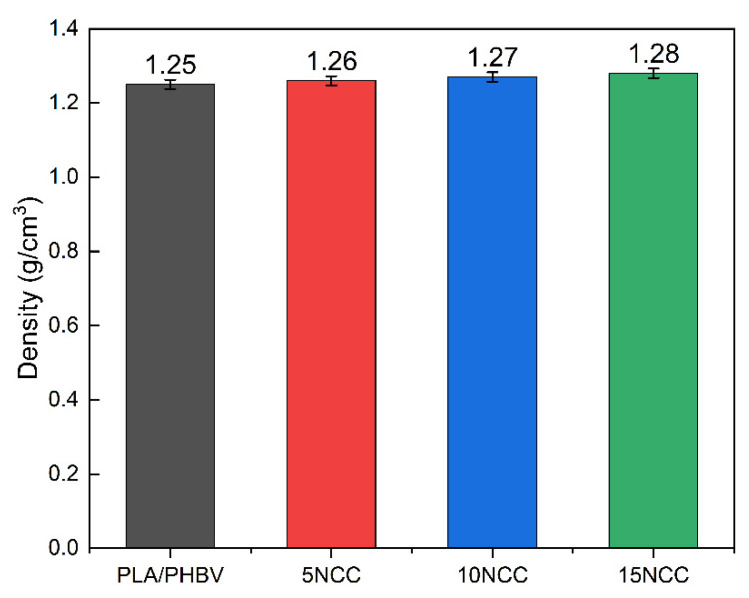
Density of produced materials.

As shown in Figure 4, the comparison of the measured density of the produced materials revealed that the composite density increased slightly with increasing filler content. This upward trend is related to the higher density of the nanocellulose used, which is 1.49 g/cm^3^, which gradually increases the density of the composite. The density of the matrix is equal to 1.24 g/cm^3^. The obtained results are correlated with theoretical density calculated based on [54]. The theoretical density of 5NCC is equal to 1.25 g/cm^3^, 10NCC 1.26 g/cm^3^, and for 15NCC 1.27 g/cm^3^.

**Figure 5:**
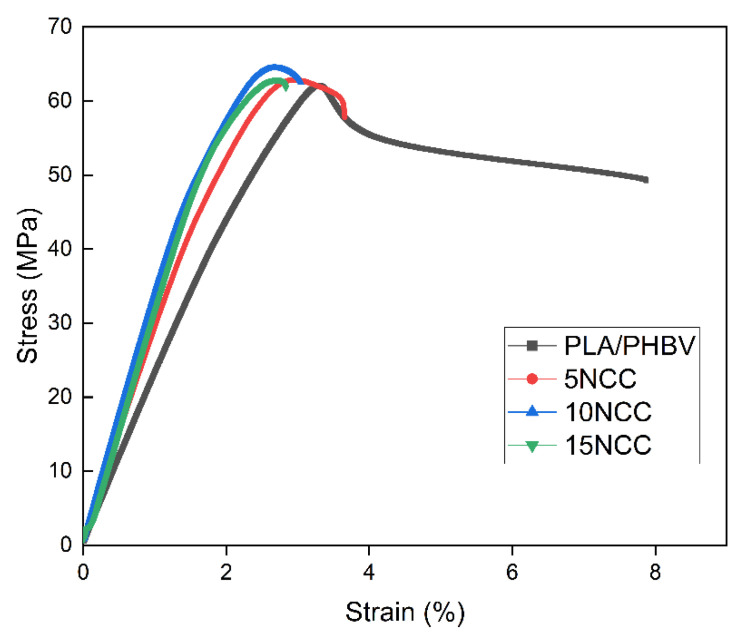
Examples of stress–strain curves of tested materials.

**Figure 6:**
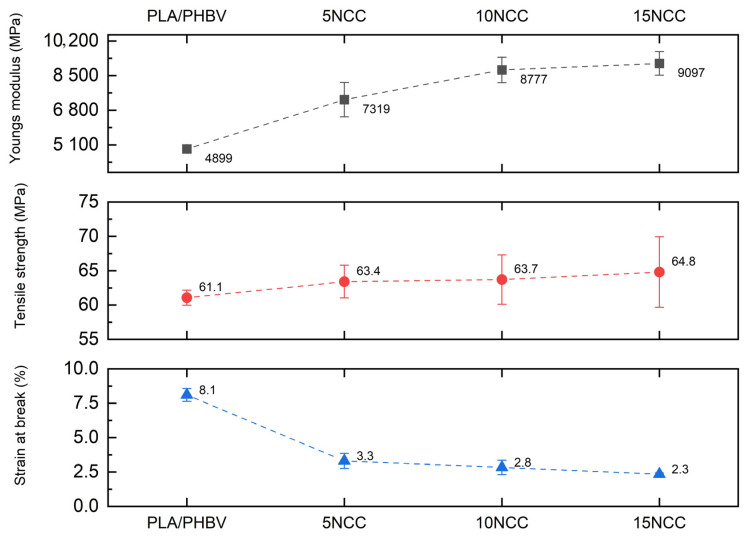
Average mechanical properties.

Figure 5 shows the tensile testing representative curves of the produced composites, whereas the average tensile properties such as strength, elastic modulus, and strain at break are compared in Figure 6.

The results of the tensile test revealed a significant reduction in sample deformation with the addition of NCC. The pure PLA/PHBV blend exhibited more than double the deformation capability compared to the composites prepared with the NCC addition. Strength property tests demonstrated that tensile strength increased with increasing NCC content, albeit within a limited range of 4–5%. Due to the substantial variability in the obtained results, it cannot be conclusively determined that the addition of nanocrystalline cellulose causes an increase in tensile strength. Furthermore, observations of fractures indicated the formation of agglomerates of introduced particles, which may potentially result in the weakening of the material. The addition of 5 wt.% NCC resulted in a 50% increase in the Young’s modulus, and 10 wt.% of NCC led to an approximate increase of 80%. A further increase in the NCC filler content yielded an increase of 85% in the elastic modulus compared to the PLA/PHBV matrix. However, as mentioned previously, the introduction of the filler significantly reduced the plastic deformation of the materials from 7% to 2.3% of the rupture strain for the composite containing 15 wt.% NCC. This behavior can be attributed to the restriction of the polymer chain deformation by the NCC particles present. A slight increase in tensile strength may be related to the formation of NCC agglomerates, which, despite ensuring high stiffness of the composite, might lead to an increase in the number of micro-notches that facilitate crack initiation [55].

#### Homogenization Procedure for the Effective Young’s Modulus

The estimation of the average properties of the new composite is an important part of the design process. Mechanical properties such as Young’s modulus may differ by an order of magnitude for the components considered in the composite. The measured value is an average of these properties, but the estimation of this property is not trivial. In the literature, there exist numerous models that provide some methods to calculate the macroscopic value of the property considered [56]. One of the first models was proposed by Voigt [57], which takes the assumption of uniform strain. Additionally, the materials considered were connected in parallel, so the relationship between the calculated property and volume fraction is linear. The model is defined as

(3) $${\bar{E}}^{V}=\overset{k}{\underset{i=0}{\sum }}\phi^{i}E^{i}$$

where ${\bar{E}}^{V}$ is an average value of Young’s modulus, $\phi^{i}$ means the volume fraction of i-th phase labeled i=0,...,k and $E^{i}$ is the Young’s modulus of i-th phase. The next model was introduced by Reuss [58] in which the materials considered are connected in series and subjected to uniform stress. This leads to a highly nonlinear relationship given by the following equation:

(4) $${\bar{E}}^{R}={\left[\overset{k}{\underset{i=0}{\sum }}\phi^{i}{\left(E^{i}\right)}^{-1}\right]}^{-1}$$

Due to the definitions of those models, they constituted the border bounds for other modern models, where the Voigt model is treated as the upper bound and the Reuss model as the bottom one. However, those models do not consider the real shape nor the orientation of the inclusions; thus, the properties of real composites vary in those models.

One of the most widely used models for estimating the average properties of short fiber-reinforced composites is the Halpin–Tsai model [59]. The effective Young’s modulus $E_{c}$ of the composite in this case takes the following form:

(5) $$E_{c}=E_{m}\left[\frac{3}{8}\left(\frac{1+\zeta \eta_{L}\phi_{f}}{1-\eta_{L}\phi_{f}}\right)+\frac{5}{8}\left(\frac{1+2\eta_{T}\phi_{f}}{1-\eta_{T}\phi_{f}}\right)\right]$$

where $\phi_{f}$ is volume fraction of reinforcing phase, $E_{m}$ is Young’s modulus of matrix, and two additional parameters $\eta_{L}$ and $\eta_{T}$, respectively, for longitudinal and transversal directions were used:

(6) $$\eta_{L}=\frac{E_{f}/E_{m}-1}{E_{f}/E_{m}+\zeta },\;\;\;\;\;\;\;\;\;\;\;\;\eta_{T}=\frac{E_{f}/E_{m}-1}{E_{f}/E_{m}+2}$$

In Equation (6) ζ is the geometrical aspect ratio introduced to calculate the longitudinal modulus and it is given by

(7) ζ=2l/d

where l and d means length and diameter of fiber, respectively.

Typically, when producing a composite, the weight proportion of the reinforcing phase is known; however, Equations (3)–(5) use information about the volume fraction, which is calculated using the following formula:

(8) $$\phi_{f}=\frac{1}{1+\frac{\rho_{f}}{\rho_{m}}\left(\frac{1}{wt}-1\right)}$$

where $\rho_{f}$, $\rho_{m}$, wt represent, respectively, the density of the reinforcing phase, matrix density, and the weight proportion of the second phase.

**Figure 7:**
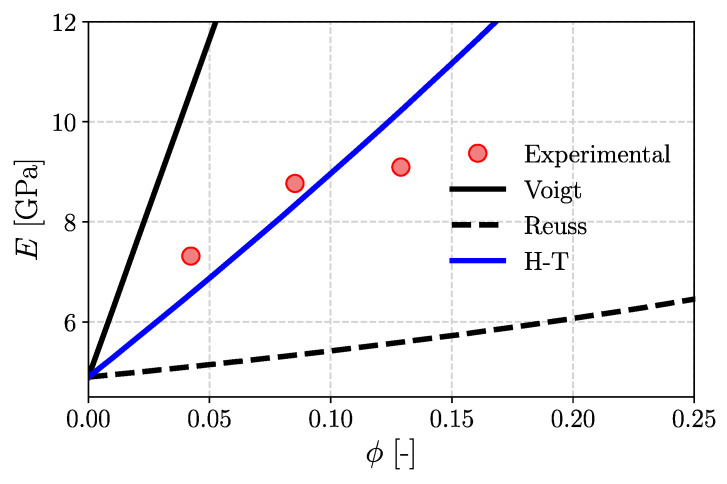
Theoretical limits of the effective Young’s modulus for composites PLA/PHBV/NCC.

Figure 7 shows a comparison of the described analytical models concerning the experimentally obtained values of Young’s modulus for the composite reinforced with NCC. As mentioned earlier, Voigt’s and Reuss’s approximations are the boundaries for experimental values and other models, Halpin–Tsai (H-T) in this case. It can be seen that the experimental results lie in the middle of the range determined by the upper and lower estimates. In the analyzed range of reinforcement volume fractions, the experimental results can be effectively described using the Halpin–Tsai model.

**Figure 8:**
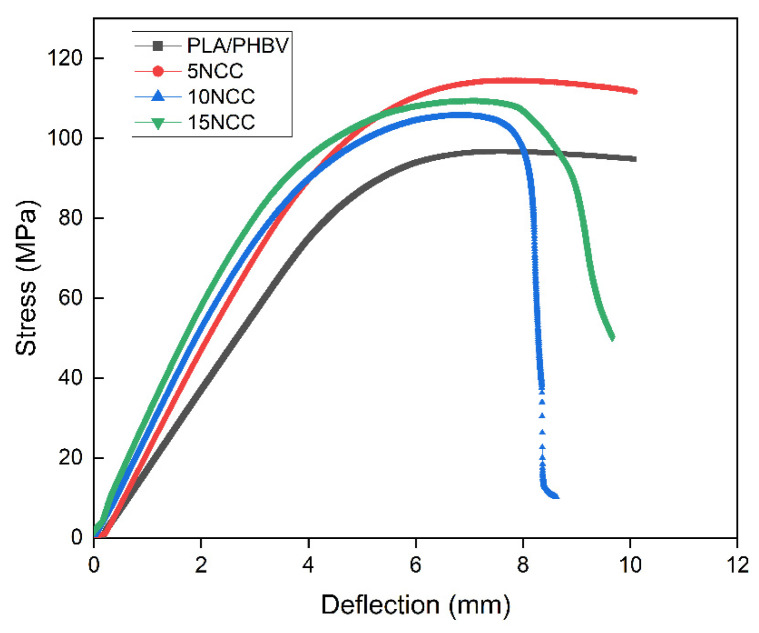
Flexural representative curves of tested materials.

**Figure 9:**
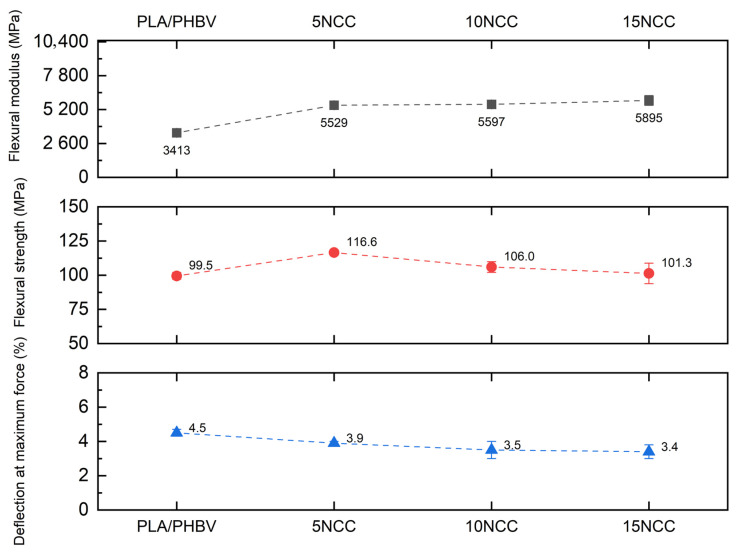
Average flexural properties.

Figure 8 provides a comparison of bending curves for the samples molded from the produced composites. It demonstrates that the addition of cellulose positively impacts the strength of the samples, but the differences in the flexural stress are small. The observed behavior of the samples and the visible differences between them are most likely attributed to the restriction of the polymer chain mobility during deformation, which limits their maximum deflection without loss of cohesion. The observed increase in stresses at smaller deformations may result from an increase in the density of the samples, as presented in the previous section. Figure 9 shows the average values of the bending strength, the bending modulus, and the deformation at the maximum force determined during a static bending test. The test results indicated that the bending strength decreased with the increase in the NCC content, while the modulus of elasticity improved significantly. The decrease in bending strength was likely associated with the formation of NCC agglomerates within the materials.

**Figure 10:**
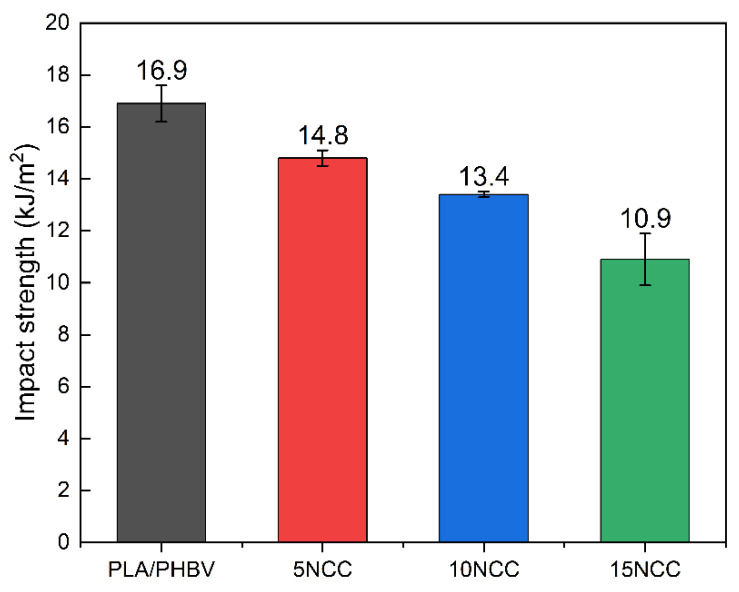
Average impact strength of molded samples.

Figure 10 shows the average impact strength and illustrates the almost linear decrease in energy required to damage the sample dynamically. Adding NCC makes the sample stiffer at the expense of impact strength, which is in line with the strength tests presented above.

Nanocrystalline cellulose (NCC) has garnered significant attention in materials science due to its unique capacity to enhance the mechanical properties of diverse composites. This effect is attributed to several key characteristics of NCC, including its high aspect ratio, large specific surface area, and superior mechanical strength. A primary factor contributing to the improvement of the mechanical properties of composites with NCC is its nanometric dimensions, which facilitate efficient stress transfer within the composite matrix. NCC, when incorporated into polymers or other matrices, functions as a reinforcing agent, substantially increasing the tensile strength and elastic modulus. Empirical studies corroborate that the addition of NCC enhances the mechanical strength of biocomposite films. Aravind et al. emphasized that the exceptional renewability and mechanical properties of NCCs are crucial factors in this enhancement [60]. Similarly, Azeredo et al. demonstrated that cellulose nanofibers in biopolymer matrices increase both the strength and modulus, albeit with a concomitant reduction in elongation [61].

The high crystallinity of NCC, obtained through acid hydrolysis, further enhances its capacity to improve mechanical properties. This process eliminates amorphous regions, resulting in a material with a tensile strength exceeding 7.5 GPa [44]. In addition to increasing the stiffness of the composite, the crystallinity of NCC also enhances thermal stability, rendering it a suitable option for applications requiring durable materials [21]. Furthermore, the rigid, rod-like structure of NCC facilitates the formation of an internal network within the matrix, which enhances load distribution and mechanical meshing [29].

The substantial specific surface area of NCC also enables superior interfacial bonding between the NCC and the matrix material. This characteristic is a crucial factor in efficient load transfer. Kasa et al. observed that modified NCC exhibited improved dispersion and interaction with polylactic acid (PLA), resulting in enhanced mechanical properties compared to unmodified NCC [40].

### 3.3. Energy Dissipation and Fatigue Examination

The composite-molded samples were then subjected to mechanical hysteresis loop tests. These tests involve low-cycle loading and enable the determination of the energy dissipated within the material during cyclic load changes. When loaded, unreinforced materials dissipate energy due to internal friction between molecules, generating thermal energy. However, this method can also be utilized to assess the molecule’s interactions within composite materials. In composite materials, the mechanical energy supplied to the system is dissipated through relaxation processes that alleviate both the processing-induced stresses and the internal stresses resulting from the introduction of reinforcement. Energy dissipation occurs through mechanisms such as pulling fiber and at the locations where defects, such as notches and discontinuities, exist [62,63].

**Figure 11:**
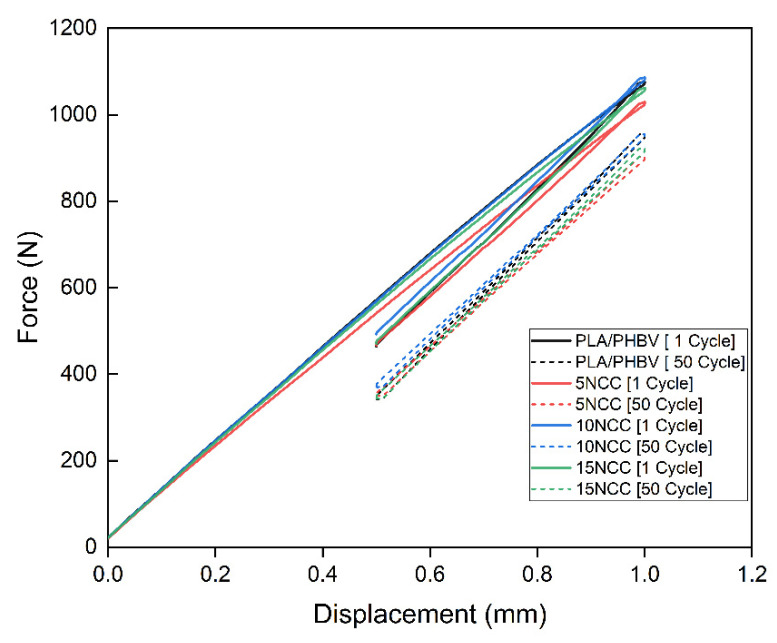
The first and fiftieth hysteresis loops determined for the molded composites.

**Figure 12:**
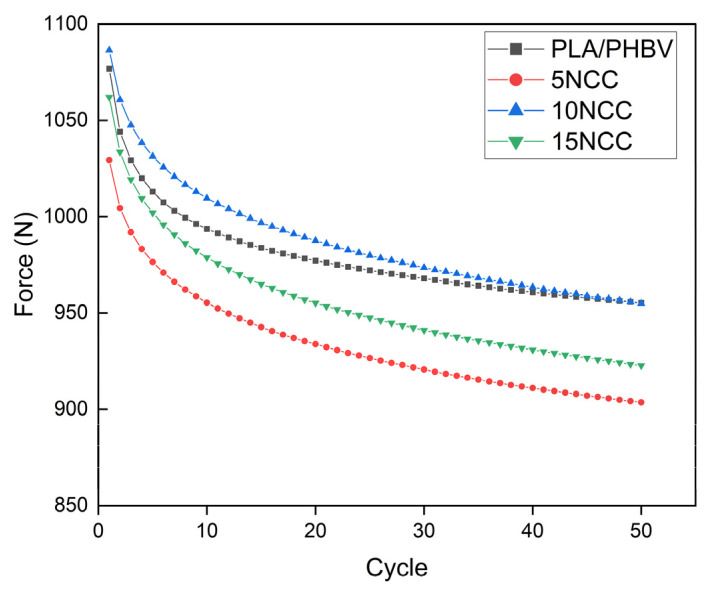
Force versus load–unload cycles of the molded composites.

Figure 11 depicts the first and fifth hysteresis loops recorded when imposing a displacement of 1 mm. The differences in the force required for the displacement are quite evident. The highest forces were recorded for the material containing 10 wt.% NCC. The forces of composites with a lower NCC content were lower compared to those of the matrix material. It was also noticed that the force needed to deform the material decreases with the increase in the number of cycles, suggesting a cyclical weakening of the materials (Figure 12).

Similar research results were presented by Porębska et al. for composite materials based on polyoxymethylene and polypropylene reinforced with glass fiber [63]. In their research, the authors presented a theory based on the analysis of energy dissipation during cyclic deformation in thermoplastic composites. They pointed to three possible effects: The first mechanism is characterized by an increase in stress with an increase in the number of cycles and a decreasing loop area, causing the material to be cyclically strengthened. The second mechanism occurs when the peak stresses decrease as the number of cycles increases until a steady state is reached; this is a cyclic material weakening mechanism. The third type indicates the stability of the material over time. The test results show that the manufactured materials exhibit the second mechanism, i.e., cyclic material weakening. Similar test results were obtained for composites such as biopolyamide reinforced with glass and basalt fibers [64], biopolyamide reinforced with basalt and aramid fibers [65], and polyoxymethylene modified with metal nanoparticles [66].

**Figure 13:**
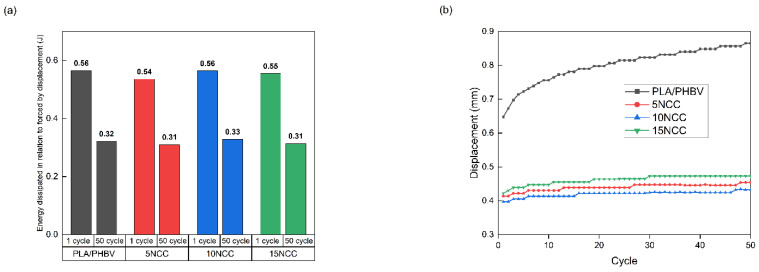
Dissipation energy (**a**) and displacement (**b**) depending on the number of load cycles.

Figure 13 shows the value of the dissipation energy and displacement depending on the filler share and the number of cycles. It can be seen that the energy dissipation in the composites is at a level similar to that of the unmodified material at the first and the fiftieth load cycles. The research results showed that the value of the dissipation energy decreases and then stabilizes after the first few cycles; this relationship can be explained on the basis of the Cieszyński and Topoliński theory. Research conducted on multiphase materials has shown that the first load cycles eliminate locally occurring stresses and material defects. The load–unload process is a kind of relaxation process that causes cracking in interphase areas that have undergone maximum stress, hence the resulting stabilization of the dissipation energy after several cycles that persists for the next several dozen cycles at an almost constant level [67]. In the case of the polymer blend, an increase in the strain was observed with an increase in the number of cycles, which suggests dynamic creep phenomena, while composite materials showed much smaller dimensional changes.

PLA/PHBV composites with the addition of NCC were subjected to accelerated fatigue tests with force excitation with a sinusoidal profile. The tests were carried out in the load–unload mode, with a frequency of 5 Hz, on a hydraulic testing machine. For all composites, the value of the minimum excitation force was unchanged during the entire test and amounted to 0.2 kN. The maximum load value in the first 5000 test cycles was 0.3 kN. Every 5000 cycles, the maximum load level was increased by 0.2 kN. The tests were carried out until the samples broke. Mechanical hysteresis loops and the maximum elongation and the maximum value of dissipation energy obtained at each load level were recorded. On this basis, in accordance with the Lehr method, fatigue strength was determined as a comparative parameter for composites tested using the same method. The relative fatigue strength was also calculated by dividing the fatigue strength value according to the Lehr method by the tensile strength value of a given composite.

**Figure 14:**
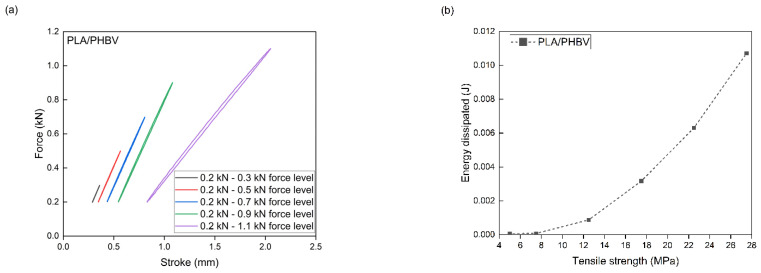
Accelerated fatigue results for polymer blend: (**a**) 5 thousandth hysteresis loop in the assumed load cycle; (**b**) the ratio of dissipation energy to tensile strength in the assumed load cycle.

**Figure 15:**
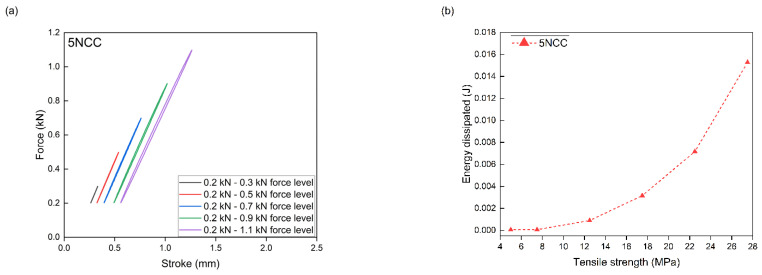
Accelerated fatigue results for polymer blend modified with 5 wt.% NCC: (**a**) 5 thousandth hysteresis loop in the assumed load cycle; (**b**) the ratio of dissipation energy to tensile strength in the assumed load cycle.

**Figure 16:**
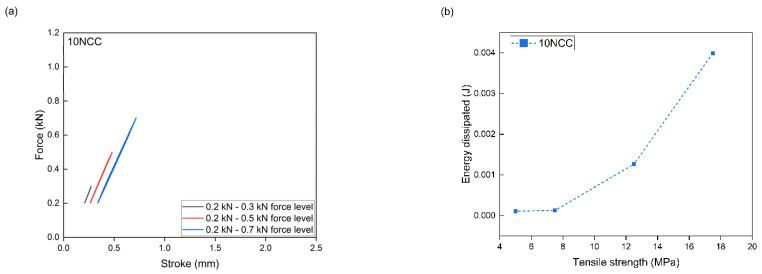
Accelerated fatigue results for polymer blend modified with 10 wt.% NCC: (**a**) 5 thousandth hysteresis loop in the assumed load cycle; (**b**) the ratio of dissipation energy to tensile strength in the assumed load cycle.

**Figure 17:**
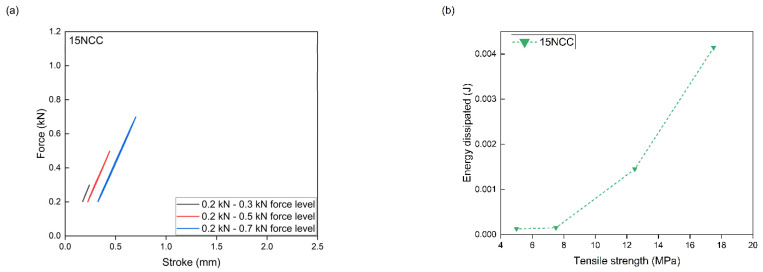
Accelerated fatigue results for polymer blend modified with 15 wt.% NCC: (**a**) 5 thousandth hysteresis loop in the assumed load cycle; (**b**) the ratio of dissipation energy to tensile strength in the assumed load cycle.

The measurement results from the accelerated fatigue tests are presented in Table 6 and Figure 14, Figure 15, Figure 16 and Figure 17. The maximum forces in the loops for an increasing number of cycles, the maximum stresses at fatigue (zz), the average elongation, and the energy released in each cycle were to be determined. The maximum transferred cyclic loads corresponding to the maximum selected load for the tested samples were 45% for PLA/PHBV, 43.4% for 5NCC, 35.3% for 10NCC, and 34.7% for 15NCC of their maximum tensile forces. The addition of NCC fibers does not increase the fatigue strength. The reason may be the heating of the viscoelastic matrix of the PLA/PHBV blend or the pulling of fibers from the material matrix during cyclic loading. The heating of the viscoelastic matrix may cause damage to the composites before breaking or pulling the fibers from the matrix. The recorded hysteresis loops presented in Figure 14, Figure 15, Figure 16 and Figure 17 are characterized by an increase in area with an increasing number of cycles. An increase in the average elongation of the sample was also observed in all tested materials. This effect can be attributed to the effects of material creep under cyclic loading. Similar test results were recorded for composites based on biopolyamide reinforced with glass, carbon, and linen fibers. This study indicates a decrease in fatigue strength after the introduction of fibers. The test results were justified by the increase in temperature during fatigue tests and fiber cracking. However, the advantages of composites were highlighted, including greater stiffness and reduced creep, which was also observed in the presented test results [68].

### 3.4. Influence of Hydrothermal and Accelerated Aging

**Figure 18:**
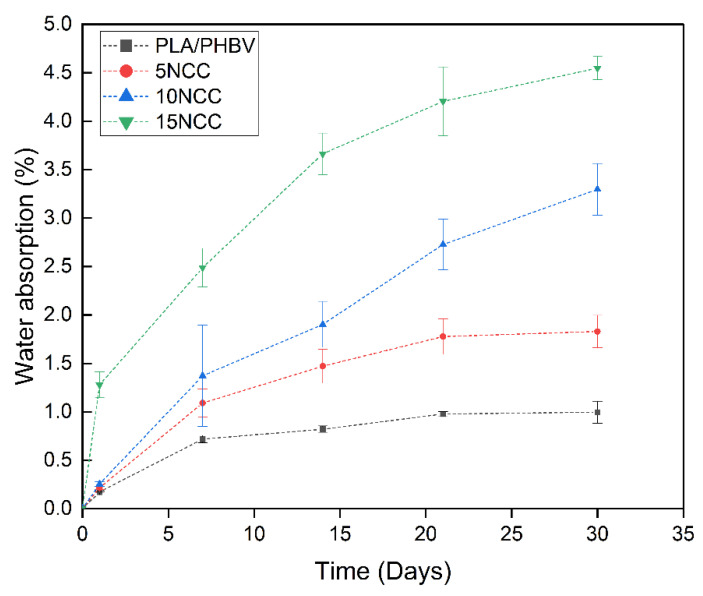
Water absorption of tested materials.

The analysis of water absorption (Figure 18) showed that water absorption increases with increasing incubation time of the samples in water. Additionally, a significant impact of the addition of nanocellulose on water absorption was noticed. The higher the cellulose content, the more water the material absorbed. In the case of samples with a content of 10 wt.% NCC, the initial stage of diffusion was very similar to the base material, and only with each subsequent measurement did the absorption value increase for samples with a higher nanocellulose content. In the case of samples with 15 wt.% NCC, the absorption was much higher from the very beginning and was much faster than in the other materials.

Natural particles and fibers are highly hygroscopic, absorbing or releasing moisture based on the fiber type, temperature, and air humidity. They aim to reach a hygroscopic equilibrium, where the water vapor pressure on their surface matches that in the air. This balance is maintained only under constant temperature and humidity, requiring controlled conditions. Normally, air temperature and humidity fluctuate daily and seasonally, altering the moisture content of these fibers and particles. This variability can lead to processing issues; during plasticization, moisture in undried particles and fibers evaporates due to high temperatures, causing foaming and potentially reducing product strength [69].

Natural fibers absorb moisture due to their hydrophilic nature, with numerous hydroxyl groups (-OH) in hemicellulose and cellulose. Lignin, having a low -OH to C ratio, imparts hydrophobic properties. High -OH to C ratios in cellulose and hemicellulose enhance water accessibility. Semi-crystalline cellulose fibers increase absorption with higher amorphous content. Water absorption in natural fibers begins with swelling as water fills spaces between microfibrils, forming a temporary microcapillary network. Water absorption occurs as bound water, which is attached to cell walls and central lamellas via hydrogen bonds, and free water, which fills cell wall pores and is held by capillary forces. Bound water saturates cell walls and central lamellae until saturation (20–40% for wood cells), after which free water saturates lumens and porosities. Water forms either a multilayer or a tightly bound monolayer with -OH groups. Factors affecting water absorption in natural fiber-reinforced polymers include fiber volume fraction, temperature, fiber nature, water circulation, cross-linking and crystallinity, diffusivity, and water–polymer interactions. Wetting informs the behavior of a material’s surface when in contact with a liquid, and fibers have varying wettability. To assess if a material is hydrophobic or hydrophilic, the contact angle of a liquid drop on its surface is measured. Natural fibers have a developed surface with numerous channels in their cross-section, while cellulose fibers feature a capillary internal structure. In contrast, mineral fibers have a simple structure with a full, smooth cross-section and minor diameter changes along their length. Mineral fibers are highly hydrophobic, exhibiting low water absorption [70,71].

Moisture absorption in cellulose fiber-reinforced polymers happens due to water dissolving in the polymer structure, hydrogen bonding with hydrophilic groups in the composite, and microcracks on the surface that transport and deposit water. Absorbed water includes both free and bound water; free water moves independently through empty spaces, whereas bound water is limited to polar polymer groups. When exposed to moisture, free water binds to the fiber’s hydrophilic groups, forming hydrogen bonds that reduce fiber–matrix adhesion. Cellulose fibers swell, increasing stress in boundary areas and causing brittleness and microcracks in the matrix around the fibers. This enhances capillarity and moisture transport, leading to fiber destruction and detachment from the matrix. Over time, biological activities like fungal growth degrade natural fibers. Thus, water absorption limits the suitability and physical and mechanical properties of the composite. Polymeric materials are frequently exposed to humid environments. Water molecules can move within the polymer, altering its physical and mechanical properties. The key factors influencing moisture sorption in polymers are their chemical composition and microstructure. Moisture diffusion in polymer composites occurs through three mechanisms: diffusion of water molecules in micro-gaps between polymer chains, moisture transfer through gaps and defects at the fiber–matrix interface due to poor initial wetting and impregnation, and transport through microcracks in the matrix from production processes. The high cellulose content in plant fibers exacerbates water penetration at the interface through fiber swelling-induced microcracks, reducing composite efficiency. Increased filler dimensions cause composite cracks, enhancing moisture transport. Higher temperatures accelerate moisture absorption, significantly reducing saturation time. In natural fiber composites, water transport through matrix microcracks from fiber swelling is particularly critical. Samples with higher fiber content show a higher diffusion coefficient due to increased water absorption and cellulose content. Microcracks at the interface, caused by fiber swelling, boost water diffusion, and the capillary mechanism facilitates water flow through the fiber–matrix interface, increasing diffusivity. Researchers found that long fibers swell more and absorb more water than short fibers and particles due to their larger size and higher water affinity. Hydrophilic natural fibers are incompatible with hydrophobic thermosetting resins; thus, chemical treatment is necessary to enhance fiber–matrix adhesion [72].

However, there are distinctions between classical cellulose and nanocrystalline cellulose that influence their properties. The disparities in water absorption between cellulose and nanocellulose are primarily attributable to their structural characteristics and the difference in the scale at which they operate. Cellulose, a polysaccharide comprising linear chains of glucose units, exhibits characteristic hydrophilicity due to the presence of numerous hydroxyl groups. Nevertheless, its capacity to absorb water is constrained by the crystal structure and degree of polymerization, which can impede the access of water to the interior of cellulose fibers [73,74]. Conversely, nanocellulose, including both cellulose nanocrystals (CNCs) and cellulose nanofibers (CNFs), possesses a significantly higher surface-to-volume ratio due to its nanoscale structure. The increased surface area facilitates more intensive interaction with water molecules through enhanced exposure of hydroxyl groups. Research has demonstrated that nanocellulose can form a gel structure when combined with water, effectively retaining water within its nanoporous network [75].

**Figure 19:**
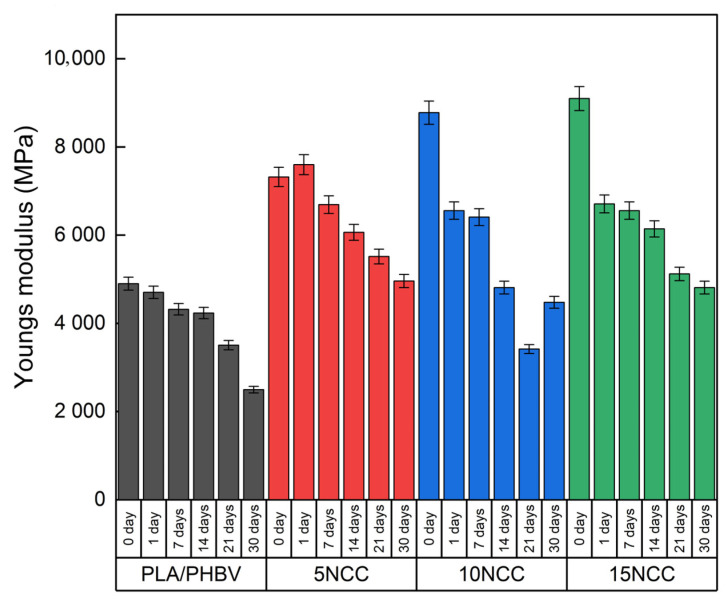
Hydrothermal effect on Young’s modulus of tested materials.

**Figure 20:**
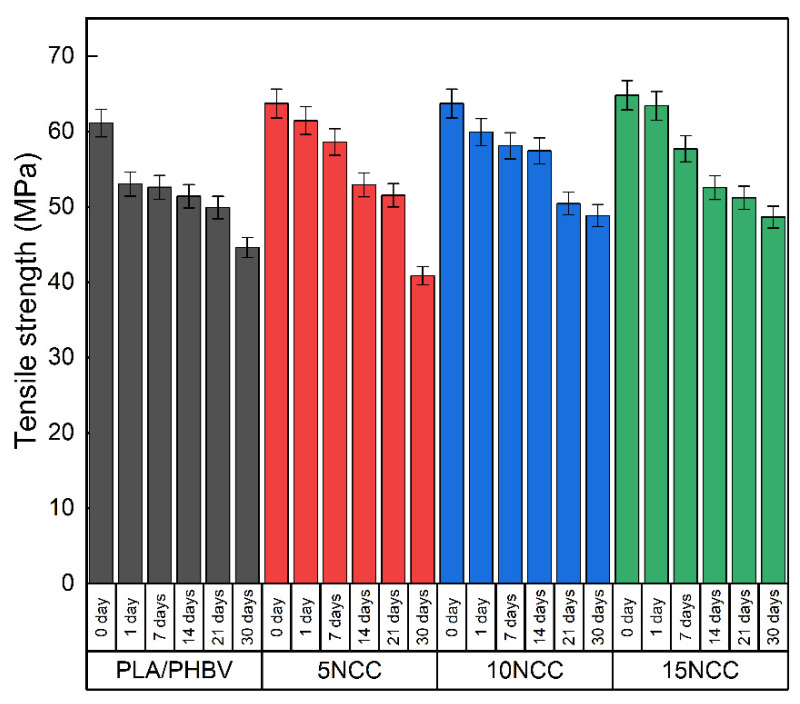
Hydrothermal effect on tensile strength of tested materials.

**Figure 21:**
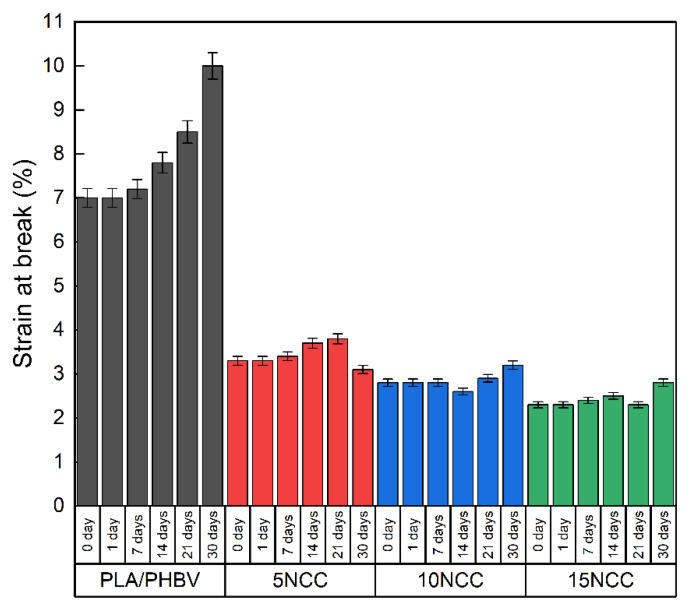
Hydrothermal effect on strain at break of tested materials.

The results of strength tests conducted after hydrothermal aging are presented in Figure 19, Figure 20 and Figure 21. There is an evident trend of increasing strain at the break with the increase in the time of exposure to the aqueous environment. The greatest increase is visible in the case of a pure blend, whereas composites exhibit a much smaller increase, indicating that the dimensional stability of the composites is maintained despite the impact of the aqueous environment.

The Young’s modulus of the polymer blend and the composite with 5 wt.% NCC has a slight decrease during the exposure to liquid, while it slightly increased after the 5 wt.% NCC sample underwent a one-day incubation in water. The remaining composites with 10 wt.% and 15 wt.% NCC content are characterized by a significant decrease in properties from the first day of exposure to the aquatic environment.

A similar relationship characterizes the tensile strength of each of the tested materials. As the incubation time increases, the tensile strength decreases. Similar values can again be observed in the case of pure materials and materials containing 5 wt.% NCC, where after 30 days, the tensile strength was approximately 40 MPa. In the case of composites with 10 wt.% and 15 wt.% NCC, after 30 days of incubation of the samples in water, the tensile strength decreased by approximately 17% from 60 MPa to approximately 50 MPa.

The above-mentioned findings can be related to cellulose and, more specifically, to the hydrophilic nature of natural fibers. Natural fibers are made of hydroxyl groups, and a high ratio (-OH) to C makes them available to water when these hydroxyl groups are exposed. Due to the semi-crystalline nature of the fibers, water absorption may vary and depends on the amount of the amorphous phase [76].

**Figure 22:**
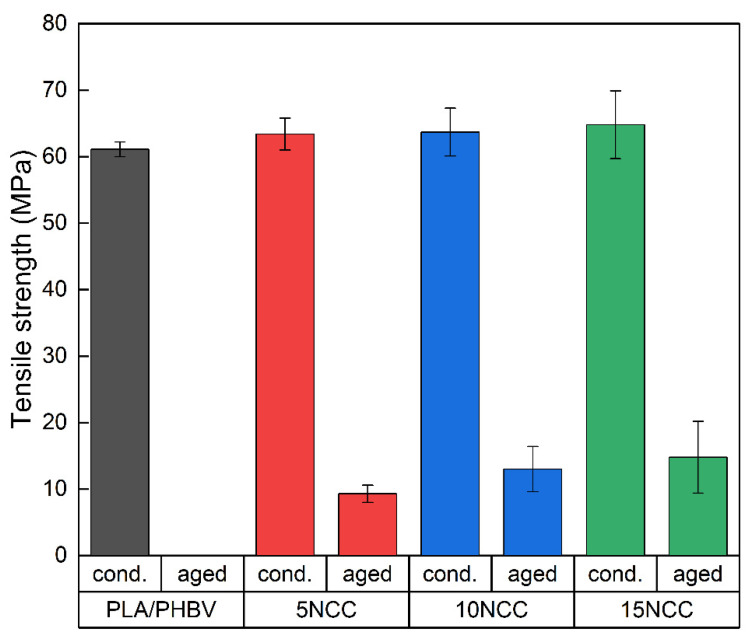
Comparison of tensile strength before and after accelerated thermal aging lasting 300 h.

**Figure 23:**
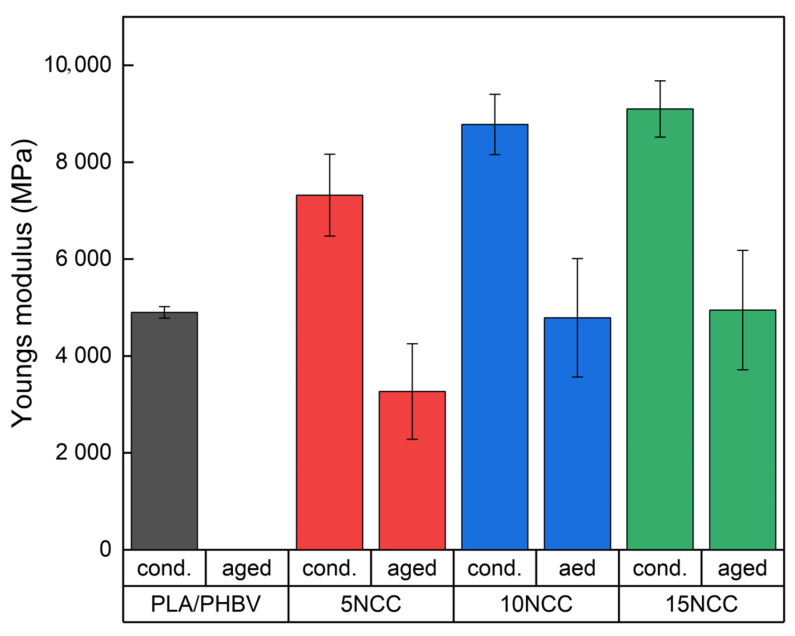
Comparison of Young’s modulus before and after accelerated thermal aging.

Visible effects of chemical changes occurring during the heating of polymers may include a reduction in molecular weight and the release of low molecular weight gaseous products. Linear polymers are characterized by chain degradation and a reduction in molecular weight. In the case of polymers with complex chain structures, side group cracking reactions also occur in addition to the polymer chain breaking process. Therefore, the stability and durability of polymers depend largely on the bond strength. The nature and energy of bonds influence both the mechanism of the degradation process and the duration of the process [77]. Strength tests were repeated after the accelerated thermal aging process. The test results are presented in Figure 22 and Figure 23.

In the case of PLA/PHBV samples, it has no value because after the aging test, the samples degraded, which made it impossible to carry out strength tests. The samples were brittle after being removed from the aging chamber. Strength tests were carried out on composites with the addition of nanocellulose, and the test results showed that NCC increases the material’s resistance to aging factors. However, the results of strength tests for materials not subjected to the aging process showed a significant impact of the applied aging parameters on the mechanical properties, causing a decrease in the tensile strength by 86%, 80%, and 77%, and the elastic modulus by 57%, 45% and 46% for the composites with a cellulose content of 5 wt.% NCC, 10 wt.% NCC, and 15 wt.% NCC. The results were obtained after 300 h of accelerated aging. The extension of the aging time resulted in the complete degradation of the materials except for the composite containing 15 wt.% nanocrystalline cellulose, which maintained its durability above 600 h.

### 3.5. Microscopic Examination

**Figure 24:**
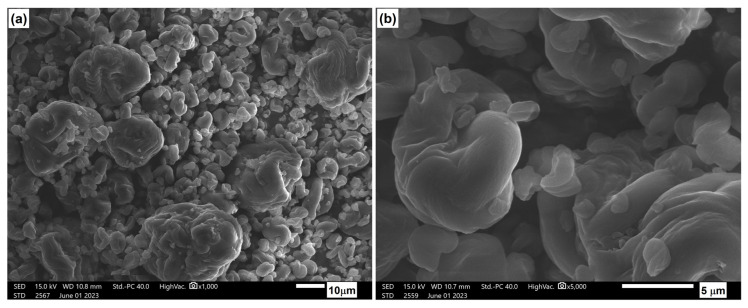
Microstructure of NCC: (**a**) 1000× magnification; (**b**) 5000× magnification.

As part of the study, the microstructure of the tested composite materials was analyzed. Figure 24 shows photos of the structure of the NCC filler used, which exhibited particle sizes ranging from nanoscale to approximately 10 μm. The geometry of the fillers is a round-like shape, although this shape is quite intricate. Notably, the cellulose walls seem to have a smooth surface, definitely different than in the case of other cellulose fillers, which are characterized by a very rough, complex, and irregular structure.

**Figure 25:**
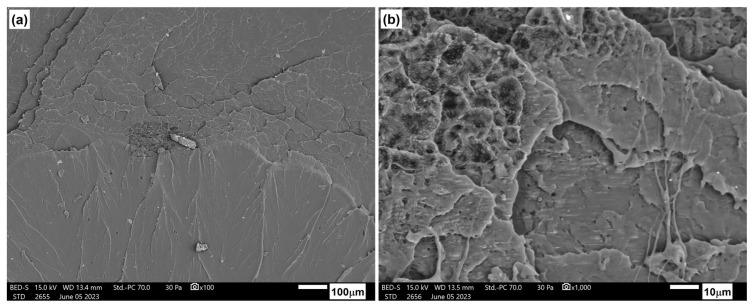
Microstructure of PLA/PHBV blend: (**a**) 100× magnification; (**b**) 1000× magnification.

Figure 25 depicts the structure of the blend of polylactide and polyhydroxyalkanoate. This material exhibits a brittle fracture with visible crystalline areas, which are responsible for the high strength properties of the tested material.

**Figure 26:**
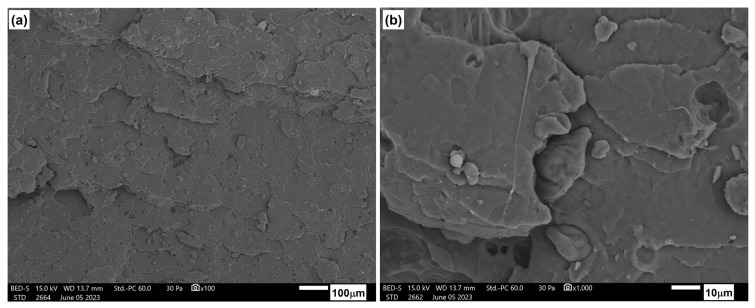
Microstructure of PLA/PHBV blend modified with 5 wt.% NNC: (**a**) 100× magnification; (**b**) 1000× magnification.

**Figure 27:**
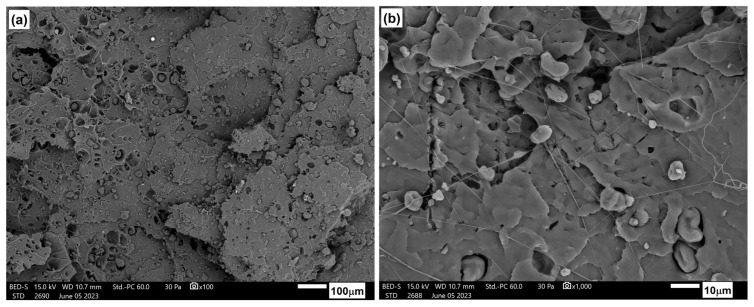
Microstructure of PLA/PHBV blend modified with 10 wt.% NNC: (**a**) 100× magnification; (**b**) 1000× magnification.

**Figure 28:**
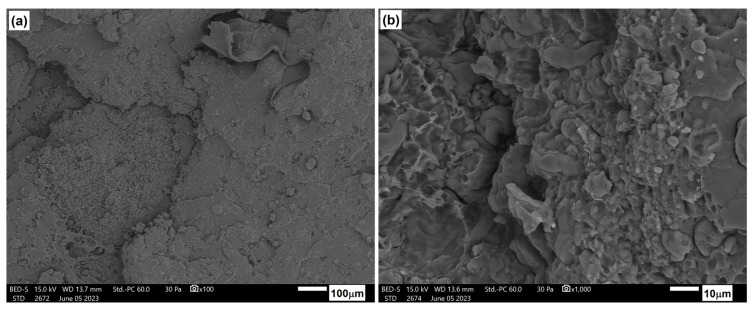
Microstructure of PLA/PHBV blend modified with 15 wt.% NNC: (**a**) 100× magnification; (**b**) 1000× magnification.

Figure 26, Figure 27 and Figure 28 show the microstructure of PLA/PHBV composites modified with NCC in amounts of 5, 10, and 15 wt.%. In each case, the structure was characterized by a brittle fracture. Cellulose particles were observed to be quite well and evenly distributed in the composite matrix. A qualitative assessment of the adhesion between the components suggests its rather moderate strength since the microscopic photos show cracks in the areas where particles were pulled out of the matrix and free spaces in the interfacial regions between the particles and the matrix. Nevertheless, the composites with a 15 wt.% NCC exhibit a slight structure change in the base material from brittle to more plastic. This is accompanied by clearly better embedded cellulose particles in the composite matrix, as confirmed by strength tests, resulting in the highest stiffness among the tested compositions.

**Figure 29:**
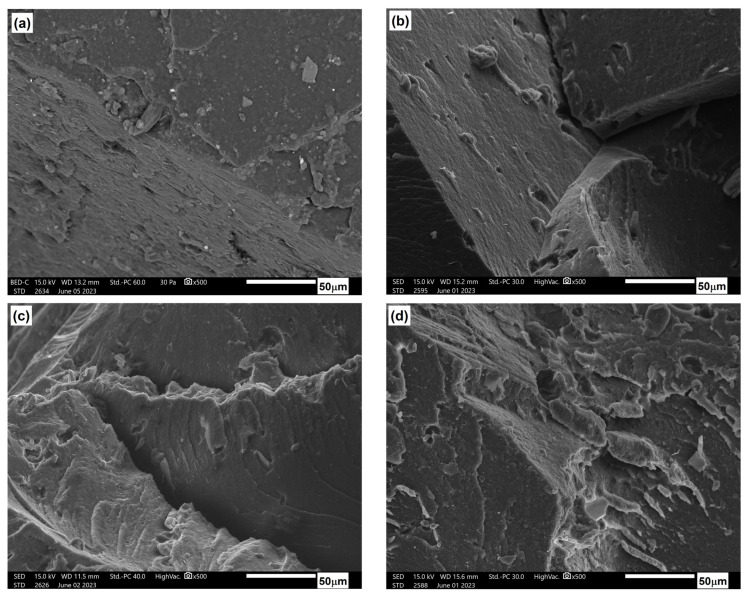
Microstructure of PLA/PHBV blend composites after 330 h of accelerated thermal aging: (**a**) PLA/PHBV, (**b**) 5NCC, (**c**) 10NCC, (**d**) 15NCC.

**Figure 30:**
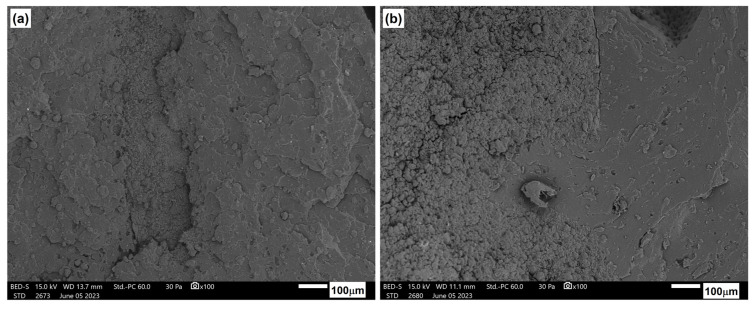
Microstructure of 15NCC: (**a**) before and (**b**) after 600 h of accelerated aging.

Figure 29 illustrates the microstructure of the tested compositions after an aging process of 330 h. In comparison to the microstructure of materials prior to the aging process, alterations in the nature of fractures are evident. These materials exhibited highly brittle fractures, sharp edges, and cracks both at the interface and within the matrix itself. Some banding was observed in the ozone structure, suggesting facile cracking between crystallites. Only samples containing 15 wt.% nanocrystalline cellulose did not undergo complete degradation after 600 h of accelerated aging. Figure 30 presents a comparison of the microstructure before and after the aging process of 600 h. Progressive degradation of the material is observable; however, preliminary results of the study indicate a positive effect of the NCC additive in limiting the degree of degradation when exposed to the external environment.

## 4. Conclusions

The findings of the research allow for several key conclusions to be drawn:

1. The incorporation of NCC results in only a minimal increase in the density of the composites relative to the matrix material;
2. Both the blend and composite materials could be characterized as amorphous materials since all materials underwent a post-crystallization process when heated above their glass transition. A slight increase in the crystallization degree with a higher NCC content was also observed, which can be considered negligible. Regardless of the NCC content, the NCC addition reduces the glass transition temperature of PLA/PHBV from around 60 °C to around 53 °C. The elastic modulus of 15NCC specimens was consistently higher, up to its own glass transition temperature;
3. While the addition of particles and agglomerates typically leads to a decrease in the tensile strength of composites, in the case of NCC, a slight increase in tensile strength was observed, along with a significant, nearly two-fold increase in stiffness in both tensile and flexural tests after the addition of 15 wt.% NCC;
4. Mechanical hysteresis loop tests showed that as the number of cycles increased to 50 load–unload cycles, the force required to deform the material decreased, indicating a cyclical weakening of the material, and accelerated fatigue tests revealed a progressive creep process with an increase in the number of cycles;
5. Tests for water absorption revealed an increase in water absorption with longer incubation times in water, and higher additive content led to higher water absorption;
6. Strength property tests after water absorption showed a decrease in the tested properties over time for the immersed samples, but these properties remained higher for the composites compared to the unmodified material;
7. The PLA/PHBV blend and composites based on it were subjected to accelerated aging, which caused complete degradation of the polymer blend, while the composites did not degrade but exhibited a significant decrease in strength properties.

In summary, the presented results indicate that the incorporation of NCC into PLA/PHBV composites leads to a modest increase in density and a reduction in the glass transition temperature while enhancing their stiffness and tensile strength, particularly at higher concentrations (15 wt%). Although the composites exhibit a gradual decrease in strength properties following fatigue and water absorption tests, they maintain superior performance compared to the base material. Aging tests demonstrate a decline in properties; however, the composites do not fully degrade, suggesting enhanced durability relative to the matrix alone. NCC improves mechanical properties but may influence the long-term stability of the material. Due to their high stiffness, materials of this nature can be utilized for small connecting elements such as screws and nuts (components that are easily misplaced) in applications with brief life cycles, such as advertising displays.

## Figures and Tables

**Table 1:** Description of the materials produced.

Designation	Description
PLA/PHBV	90:10 PLA/PHBV as the base material
5NCC	PLA/PHBV blend with 5 wt.% NCC
10NCC	PLA/PHBV blend with 10 wt.% NCC
15NCC	PLA/PHBV blend with 15 wt.% NCC

**Table 2:** Injection molding parameters to produce testing specimens.

Temperature (°C)							Injection Pressure (bar)	Compression Pressure (bar)	Press Time (s)
Feed Zone	Zone 1	Zone 2	Zone 3	Zone 4	Nozzle	Mold			
40	180	180	185	190	185	30	1000	800	8

**Table 3:** Characteristics of the aging cycle.

Cycle	**Function**	**Intensity (W/m^2^/nm)**	**Temp. (°C)**	**Time (min)**
	UV light	1.55	60	8:00
	Water spraying	-	-	0:15
	Condensation	-	50	3:45

**Table 4:** DSC measurement results of the PLA/PHBV and PLA/PHBV/NCC molded specimens.

	NCC Content	T_g_	T_m_	ΔH_cc_	ΔH_m_	Crystallization Degree
	(%)	(°C)	(°C)	(J/g)	(J/g)	(%)
PLA/PHBV	0	56.92	158.24	25.77 ± 0.55	33.09 ± 3.04	7.87
5NCC	5	56.24	157.05	25.79 ± 2.40	31.28 ± 2.97	5.90
10NCC	10	56.45	159.00	24.84 ± 2.55	30.49 ± 4.69	6.07
15NCC	15	57.46	160.58	22.51 ± 1.47	32.80 ± 5.74	11.07

**Table 5:** DMA measurement results of the PLA/PHBV and PLA/PHBV/NCC specimens.

	NCC Content	T_g_	Elastic Modulus E′ at Different Temperatures			
			20 (°C)	30 (°C)	40 (°C)	50 (°C)
	(%)	(°C)	(MPa)	(MPa)	(MPa)	(MPa)
PLA/PHBV	0	59.95	2024	2001	1940	1827
5NCC	5	52.77	2070	1989	1903	1476
10NCC	10	52.96	2085	2008	1918	1424
15NCC	15	52.40	2183	2097	2030	1685

**Table 6:** The level of maximum forces in hysteresis loops versus the increasing number of cycles and results of calculations: maximum stress at fatigue (Zz), tensile strength (σM), relative fatigue strength (relative to fatigue strength Zz/σM × 100%) mean elongation, energy dissipated in each cycle for tested composites.

Samples	Maximum Force (kN)	Number of Cycles	Zz (MPa)	σM (MPa)	Zz/σM × 100 (%)
PLA/PHBV	1.1	27,729	10.1	61.1	16.53
5NCC	1.1	27,329	9.98	63.4	15.74
10NCC	0.9	15,850	5.17	63.7	8.12
15NCC	0.9	15,449	4.77	64.8	7.36
The chosen level of Pmax (kN)	0.3	0.5	0.7	0.9	1.1
Number of cycles	5000	10,000	15,000	20,000	25,000
PLA/PHBV					
Maximum force in loop (kN)	0.29	0.50	0.69	0.90	1.11
Mean elongation (mm)	0.29	0.56	0.80	1.08	2.15
Energy dissipated in each cycle	0.000079	0.00087	0.0031	0.00730	0.01070
5NCC					
Maximum force in loop (kN)	0.29	0.49	0.69	0.90	1.09
Mean elongation (mm)	0.33	0.53	0.76	1.02	1.26
Energy dissipated in each cycle	0.000068	0.00090	0.00316	0.00717	0.01527
10NCC					
Maximum force in loop (kN)	0.29	0.49	0.70	0.56 *	-
Mean elongation (mm)	0.27	0.48	0.71	0.61 *	-
Energy dissipated in each cycle	0.00013	0.00126	0.00399	0.00048 *	-
15NCC					
Maximum force in loop (kN)	0.29	0.49	0.70	0.52 *	-
Mean elongation (mm)	0.17	0.44	0.69	0.57 *	-
Energy dissipated in each cycle	0.00014	0.00144	0.00414	0.00006 *	-

* Breaking the sample.

## Data Availability

The raw data supporting the conclusions of this article will be made available by the authors on request. The data are not publicly available due to University does not provide public open repository.
